# Artificial Intelligence in Cardiovascular Imaging: From Automated Acquisition to Precision Diagnostics and Clinical Decision Support

**DOI:** 10.3390/medsci14010132

**Published:** 2026-03-11

**Authors:** Minodora Teodoru, Alexandra-Kristine Tonch-Cerbu, Dragoș Cozma, Cristina Văcărescu, Raluca-Daria Mitea, Florina Batâr, Horea-Laurentiu Onea, Florin-Leontin Lazăr, Alina Camelia Cătană

**Affiliations:** 1 County Clinical Emergency Hospital of Sibiu, 550245 Sibiu, Romania; minodora.teodoru@ulbsibiu.ro (M.T.); onea.horea.laurentiu@elearn.umfcluj.ro (H.-L.O.); lazar.florin.leontin@elearn.umfcluj.ro (F.-L.L.); alinacamelia.catana@ulbsibiu.ro (A.C.C.); 2 Medical Clinical Department, Faculty of Medicine, “Lucian Blaga” University, 550024 Sibiu, Romania; daria.mitea@ulbsibiu.ro; 3 Institute of Cardiovascular Diseases Timisoara, 300310 Timisoara, Romania; dragos.cozma@umft.ro (D.C.); cristina.vacarescu@umft.ro (C.V.); 4 Cardiology Department, “Victor Babeș” University of Medicine and Pharmacy, 300041 Timisoara, Romania; 5 Research Center of the Institute of Cardiovascular Diseases Timisoara, 300310 Timisoara, Romania; 6 Department Basic Science-Physiology, Faculty of Medicine, “Lucian Blaga” University, 550024 Sibiu, Romania; florina.batar@ulbsibiu.ro; 7 Medical Clinic Number 1, 4th Department of Internal Medicine, “Iuliu Haţieganu” University of Medicine and Pharmacy, 400023 Cluj-Napoca, Romania

**Keywords:** artificial intelligence, cardiovascular imaging, echocardiography, cardiac magnetic resonance, computed tomography, nuclear cardiology, precision medicine

## Abstract

Cardiovascular imaging is a cornerstone of modern cardiology, yet its clinical impact is limited by operator dependence, inter-observer variability, time-consuming workflows, and unequal access to advanced expertise. Artificial intelligence (AI), particularly machine learning and deep learning, offers new opportunities to overcome these limitations. This review aims to summarize current and emerging AI applications in cardiovascular imaging and to evaluate their potential clinical value in precision diagnostics and decision support. This narrative review synthesizes clinically relevant literature on AI applications across major cardiovascular imaging modalities, including echocardiography, cardiovascular magnetic resonance, cardiac computed tomography, and nuclear cardiology. Evidence was analyzed with a focus on AI-enabled acquisition support, image segmentation, quantitative and functional assessment, workflow automation, and risk stratification, alongside key methodological and implementation considerations. Across imaging modalities, AI-driven approaches have demonstrated improved reproducibility, efficiency, and scalability of cardiovascular imaging workflows. Automated algorithms reduce operator dependence, facilitate standardized extraction of imaging biomarkers, and support advanced functional assessment and prognostic stratification. Recent developments in video-based, temporal, and multimodal models further expand AI capabilities from technical automation toward integrated disease phenotyping and personalized clinical decision support. However, translation into routine practice remains limited by heterogeneous datasets, insufficient external validation, algorithmic bias, limited interpretability, and challenges related to regulatory approval and workflow integration. Artificial intelligence has the potential to reshape cardiovascular imaging into a more efficient, reproducible, and patient-centered precision medicine tool. Real-world clinical impact will depend on outcome-driven evaluation, robust external validation, multimodal data integration, and human-in-the-loop implementation strategies that ensure safe, equitable, and clinically meaningful adoption.

## 1. Introduction

### 1.1. Cardiovascular Imaging: Clinical Value and Persistent Workflow Limitations

Cardiovascular imaging is central to modern cardiology, supporting diagnosis, risk stratification, and management across a broad spectrum of cardiovascular disease. However, despite rapid technological expansion (echocardiography, CMR, CT, and PET), much of the evidence base remains observational, raising ongoing questions regarding appropriateness, timing, and outcome impact of specific imaging strategies. As healthcare systems shift toward value-based care, strengthening outcome-driven evidence and harmonizing imaging practice remain key priorities [1].

Recent advances have expanded both technological capabilities and clinical applications. Updated reference values and AI-assisted quantification refine echocardiography; novel CMR sequences and advanced tissue characterization improve non-invasive hemodynamic and diagnostic assessment; and CT-derived calcium scoring and plaque phenotyping increasingly support ischemic heart disease risk stratification and pre-procedural planning, including structural interventions. Across ischemic heart disease, valvular disease, cardiomyopathies, myocarditis, and heart failure, imaging biomarkers such as strain, LGE, and parametric mapping contribute to more personalized management [2]. Imaging therefore represents a cornerstone of precision cardiology, enabling integrated characterization of anatomy, function, and disease biology, particularly when combined with biomarkers and genetic information [3,4].

Despite these advances, cardiovascular imaging remains constrained by operator dependence, variability, and time-intensive post-processing. Even widely used measurements such as LVEF show substantial interobserver variability in echocardiography. Large multicenter studies demonstrate that automated echocardiographic pipelines can improve feasibility and reproducibility for LVEF and GLS while maintaining prognostic performance comparable to expert interpretation and CMR reference standards [5,6]. Real-world comparisons with same-day CMR further support improved agreement and reduced misclassification using AI-driven automation, particularly in impaired function or suboptimal image quality [7]. Operator-related variability also affects CMR, where structured training improves reproducibility of volumetric and functional measurements, emphasizing that expertise remains critical even in highly standardized modalities [8]. Collectively, these limitations create workflow bottlenecks and contribute to variability in clinical decision-making, motivating the adoption of scalable automation and decision-support solutions [9].

### 1.2. Current Unmet Needs: Standardization, Efficiency, and Access to Expertise

Persistent challenges across modalities include limited standardization, long processing times, and uneven access to advanced expertise. In echocardiography, automated LVEF approaches demonstrate high feasibility and strong agreement with expert assessment, highlighting the potential of automation to reduce variability compared with conventional methods [10]. Similarly, CMR remains vulnerable to inter- and intra-operator variability, which can be mitigated through dedicated training and standardized acquisition/post-processing protocols [11]. Beyond technical advances, methodological heterogeneity and the lack of large, prospective evidence remain major obstacles to evidence-based cardiovascular imaging, potentially contributing to inconsistent clinical impact and overuse [12].

A major barrier to broader adoption of advanced imaging is workflow inefficiency. Manual contouring and tissue characterization in CMR and advanced analyses in CT (e.g., quantitative plaque assessment, CT-FFR, calcium scoring) remain time-consuming and operator-dependent, potentially delaying clinical decisions in high-volume settings [13,14]. In parallel, advanced multimodality expertise is concentrated in tertiary centers, creating geographic and institutional disparities in access and interpretation quality. As imaging complexity increases, the gap between technological capabilities and available expertise is likely to widen, underscoring the need for scalable automation, decision-support tools, and standardized training frameworks [15,16].

### 1.3. How Artificial Intelligence Can Address These Challenges

AI offers a practical framework to reduce operator dependence, accelerate analysis, and expand access to high-quality interpretation. Across echocardiography, CMR, CT, and nuclear imaging, reviews consistently report improved automation, quantitative assessment, and risk stratification, supporting more efficient and personalized cardiovascular care [2,17,18]. A key contribution is automation of labor-intensive tasks (segmentation, contouring, plaque quantification), reducing post-processing time and inter-operator variability while enabling scalable extraction of imaging biomarkers [17,18].

Clinical studies provide proof of concept: EchoNet-Dynamic demonstrated robust video-based deep learning for beat-to-beat LV segmentation and LVEF estimation, supporting standardized echocardiographic quantification [19,20]. In ischemic heart disease, machine-learning analyses integrating clinical and test data can improve prediction of CAD on coronary CT angiography compared with conventional risk scores, with potential to optimize referral pathways and resource allocation. Deep learning applied to CCTA can identify functionally significant stenosis by analyzing myocardial and coronary patterns, supporting non-invasive functional triage against invasive reference standards [21,22].

Overall, AI is increasingly positioned as an enabling technology across the imaging pipeline from acquisition support and reconstruction to quantification and clinical integration, helping address variability, workflow inefficiency, and limited access to advanced expertise. The following sections summarize core AI methodologies and their clinical applications across major imaging modalities.

**Figure 1:**
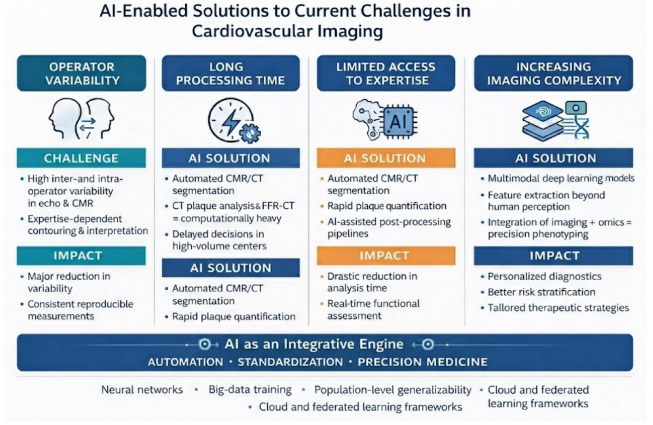
The main AI-driven strategies that overcome current challenges in cardiovascular imaging.

An overview of how artificial intelligence addresses key limitations in contemporary cardiovascular imaging is illustrated in Figure 1.

### 1.4. Core AI Methodologies in Cardiovascular Imaging

Artificial intelligence in cardiovascular imaging encompasses a spectrum of machine learning (ML) and deep learning (DL) approaches, each suited to different types of data and clinical tasks. Traditional ML methods rely on predefined feature extraction and are often applied to structured clinical variables, whereas deep learning enables automated representation learning directly from raw imaging data. Owing to the high dimensionality and complexity of cardiovascular images, deep learning has become the dominant paradigm for image-based applications [23,24,25,26].

Convolutional neural networks (CNNs) form the methodological backbone of most current imaging systems. Architectures such as U-Net and its derivatives enable accurate pixel-wise segmentation and have been widely adopted for automated chamber delineation, plaque characterization, and tissue analysis across echocardiography, CMR, CT, and nuclear imaging [27,28]. By learning hierarchical spatial features, CNNs reduce operator dependency and improve reproducibility in tasks that were previously labor-intensive and highly expertise-dependent.

Because many cardiovascular imaging modalities generate temporal data, including echocardiographic videos and cine CMR, sequential and video-based models have emerged to capture dynamic cardiac motion. Recurrent neural networks, long short-term memory (LSTM) models, and more recently transformer-based architectures extend static CNN frameworks by incorporating temporal dependencies and long-range contextual relationships [19,29,30,31,32]. These approaches enable automated assessment of cardiac function, view classification, and motion analysis, supporting scalable interpretation of video-based datasets.

Beyond single-modality analysis, multimodal AI models integrate imaging data with clinical variables, electrocardiography, biomarkers, and other structured or unstructured inputs. Such integrative approaches enhance disease phenotyping and risk prediction compared with isolated imaging models, particularly in complex conditions such as coronary artery disease and cardiomyopathies [33,34,35]. However, translation into clinical practice remains limited by heterogeneity of data sources and insufficient external validation.

Robust training, validation, and generalizability remain fundamental prerequisites for safe clinical deployment. Model performance must be assessed using task-specific metrics and externally validated across institutions, populations, and imaging protocols to mitigate overfitting and dataset shift [36,37,38,39]. Transparency, interpretability, and lifecycle-based regulatory oversight are increasingly recognized as essential components of responsible AI implementation in cardiovascular imaging.

**Figure 2:**
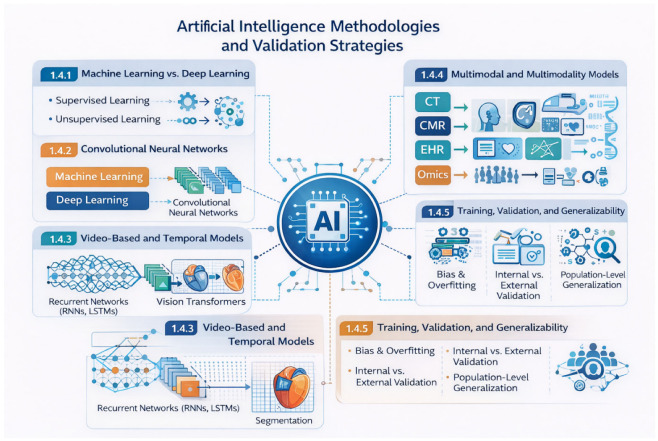
Core artificial intelligence methodologies in cardiovascular imaging.

An integrated schematic of the principal artificial intelligence methodologies and validation pathways relevant to cardiovascular imaging is shown in Figure 2.

## 2. Scope and Methods

This narrative review was informed by a structured (non-systematic) literature search conducted in PubMed/MEDLINE, Scopus, and Web of Science. The search was performed between November 2025 and February 2026, with the last update in February 2026, and focused primarily on publications from 2015 onward, reflecting the rapid evolution of artificial intelligence (AI) in cardiovascular imaging.

Search terms were combined using Boolean operators and included: (“artificial intelligence” OR “machine learning” OR “deep learning”) AND (“cardiovascular imaging” OR “echocardiography” OR “cardiac CT” OR “cardiac computed tomography” OR “cardiovascular magnetic resonance” OR “cardiac MRI” OR “nuclear cardiology” OR “SPECT” OR “PET”) AND (“segmentation” OR “ejection fraction” OR “strain” OR “FFR” OR “plaque” OR “calcium scoring” OR “tissue characterization” OR “reconstruction” OR “workflow” OR “validation” OR “generalizability” OR “bias” OR “fairness” OR “clinical implementation”). Reference lists of key articles, state-of-the-art reviews, and consensus statements were also screened to identify additional relevant publications.

Eligible publications were limited to English-language articles and included original research, multicenter or externally validated studies, major narrative or state-of-the-art reviews, and consensus/guideline documents with direct relevance to clinical cardiovascular imaging. Priority was given to studies demonstrating external validation, multi-vendor applicability, clinically interpretable outcomes, or explicit focus on translational and implementation aspects. Case reports, non-cardiovascular imaging studies, and purely technical publications without clear clinical applicability were excluded.

Study selection was performed by the authors based on relevance to the predefined scope, with the aim of synthesizing areas of convergence, identifying well-established applications, and highlighting key limitations and evidence gaps across imaging modalities.

## 3. Clinical Applications of AI in Cardiovascular Imaging

Artificial intelligence applications in cardiovascular imaging have evolved heterogeneously across modalities and clinical tasks. In this section, we adopt a synthesis-oriented framework rather than a study-by-study exposition. For each imaging modality, we first delineate domains that have reached relative technical maturity (e.g., workflow-enabling automation and externally validated quantitative tasks), followed by emerging applications that may alter clinical decision-making but require further validation. We then highlight areas of convergence across independent investigations, identify persistent methodological limitations, and discuss barriers to clinical translation.

This structured approach allows a clearer differentiation between well-established applications (characterized by reproducibility gains, multicenter validation, and integration into existing workflows) and decision-altering systems that remain in earlier developmental stages. By explicitly contrasting maturity, generalizability, and translational readiness, we aim to provide a consolidated view of the current state of the art rather than an isolated description of individual studies.

### 3.1. Echocardiography

#### 3.1.1. Classification and Acquisition Support

Automated view classification represents one of the earliest and most technically mature applications of artificial intelligence (AI) in echocardiography. Deep learning models trained on large real-world datasets have demonstrated expert-level performance in video-based view recognition across diverse acquisition conditions, significantly reducing operator dependence and enabling standardized downstream analysis. These systems improve workflow efficiency by supporting automated labeling, quality control, and acquisition optimization.

Across studies, convergence is evident regarding the robustness of view classification across vendors and pathologies, suggesting that this task has reached a relatively stable and generalizable stage of development. Nevertheless, performance remains influenced by dataset composition and acquisition heterogeneity, underscoring the importance of diverse training cohorts and rigorous external validation [31,40,41].

#### 3.1.2. LV Function: Automated EF and Segmentation from Echo Videos

Among echocardiographic applications, automated segmentation and ejection fraction (EF) estimation represent the most extensively validated domains. EchoNet-Dynamic demonstrated near–expert-level performance for beat-to-beat left ventricular segmentation and EF prediction directly from full echocardiographic sequences, with external validation confirming improved reproducibility compared with manual interpretation [19]. Subsequent prospective evaluation further supported the clinical feasibility of AI-assisted ventricular function assessment, demonstrating performance comparable to experienced sonographers with reduced variability [42].

Convergence across independent studies indicates that automated EF estimation consistently approaches expert-level accuracy, particularly in moderate-to-severe systolic dysfunction. In addition, large-scale video-based models have enabled automated extraction of imaging biomarkers associated with incident heart failure, atrial fibrillation, myocardial infarction, and mortality, expanding the role of AI beyond workflow acceleration toward scalable phenotyping [43].

However, important limitations remain. Most models are derived from retrospective datasets, and segmentation errors may occur in anatomically complex cases or suboptimal acoustic windows. While automation clearly improves reproducibility and efficiency, its incremental impact on therapeutic decision-making and long-term outcomes remains insufficiently validated in prospective, outcome-driven trials.

#### 3.1.3. Beyond EF: Automated Structure/Function Quantification and Outcomes

Recent deep learning frameworks extend automated analysis beyond EF toward quantification of chamber dimensions, wall thickness, atrial size, and strain-derived metrics. Large multi-institutional studies have demonstrated that AI-derived structural and functional parameters independently predict adverse cardiovascular outcomes, including heart failure, atrial fibrillation, myocardial infarction, and mortality [44].

Despite these promising findings, prognostic modeling remains more heterogeneous than segmentation tasks. Variability in feature engineering, cohort selection, and validation methodology limits direct comparability across studies. External multicenter validation and prospective evaluation of incremental clinical benefit remain limited, highlighting the need for standardized validation frameworks before routine deployment of decision-altering systems.

#### 3.1.4. Robustness/Fairness/Generalizability

Technical accuracy alone does not guarantee equitable or generalizable deployment. Performance variability across demographic subgroups, acquisition settings, and institutional protocols highlights the need for representative datasets and bias-aware evaluation strategies. Domain shift across centers remains a major barrier to generalizability, and transparent reporting standards are essential to ensure safe implementation in diverse clinical environments.

Collectively, AI applications in echocardiography have reached relative maturity for workflow-enabling tasks such as view classification, ventricular segmentation, and EF estimation, with consistent evidence of improved reproducibility, efficiency, and external validation. In contrast, decision-altering systems, particularly those focused on advanced prognostic modeling remain in earlier stages of development and require robust prospective validation before widespread clinical adoption [37,40].

Thus, while echocardiography represents one of the most advanced domains for AI implementation in cardiovascular imaging, sustained clinical impact will depend on outcome-driven studies, standardized validation frameworks, and careful integration into routine clinical workflows.

### 3.2. Cardiovascular Magnetic Resonance (CMR)

#### 3.2.1. Automated Segmentation & Volumetric Analysis

Automated segmentation and volumetric quantification represent the most mature applications of artificial intelligence in cardiovascular magnetic resonance (CMR). Deep learning architectures, particularly fully convolutional networks and U-Net-based models, have consistently achieved expert-level performance for left and right ventricular segmentation, enabling reproducible assessment of ventricular volumes, myocardial mass, and ejection fraction at scale [45].

Large-scale validation studies have demonstrated that fully automated pipelines can analyze complete cine CMR examinations within seconds while maintaining accuracy comparable to inter-observer variability among expert readers [46]. Benchmarking initiatives such as the Automatic Cardiac Diagnosis Challenge (ACDC) further confirmed that deep learning approaches outperform traditional multi-atlas and deformable model techniques, establishing automated segmentation as a clinically viable and scalable solution [47].

Despite this maturity, limitations persist, particularly in basal and apical slice delineation, right ventricular contouring, and robustness across heterogeneous acquisition protocols. External multicenter validation and systematic assessment of anatomical plausibility remain critical for reliable deployment in routine practice.

#### 3.2.2. Cine-CMR and Functional Assessment (EF, Strain, Motion)

Cine CMR provides an ideal substrate for AI-driven functional analysis due to standardized acquisition protocols and high spatial resolution. Deep learning–based segmentation enables consistent extraction of functional biomarkers and supports automated disease classification derived from cine-based features [47].

More recent externally validated frameworks integrate segmentation, automated quality control, and biomarker extraction within scalable clinical pipelines capable of processing large, heterogeneous datasets across multiple vendors and institutions [48]. Such systems reduce operator dependence and unlock previously underutilized clinical CMR repositories for longitudinal and population-level research.

Although cine-derived functional quantification is technically mature, evaluation metrics may not fully capture clinically meaningful contour errors. Robust validation across diverse disease phenotypes and acquisition settings remains essential before universal automation can be endorsed.

#### 3.2.3. Tissue Characterization: LGE, Mapping (T1/T2/ECV)

Tissue characterization constitutes a defining strength of CMR and an expanding domain for AI integration. Deep learning–based segmentation of late gadolinium enhancement (LGE) images enables automated infarct quantification with performance approaching expert interpretation, substantially reducing manual post-processing burden [49].

Similarly, AI-assisted segmentation of native T1 and T2 maps improves reproducibility of myocardial tissue quantification and enhances disease discrimination by integrating multiple quantitative features rather than relying on single-threshold approaches [50]. These methods facilitate more nuanced phenotyping of ischemic, inflammatory, and infiltrative cardiomyopathies.

In parallel, deep learning–based reconstruction strategies improve image fidelity and support accelerated acquisition for cine, LGE, and parametric mapping sequences, mitigating traditional trade-offs between scan time and spatial resolution [51].

#### 3.2.4. Workflow Acceleration and Reconstruction

Workflow acceleration through AI-driven reconstruction represents a cornerstone of modern CMR innovation. Model-based deep learning approaches and variational networks integrate learned priors with physics-based constraints, enabling accelerated acquisition with improved structural fidelity and reduced artifact burden compared with conventional parallel imaging and compressed sensing techniques [52].

CMR-specific deep learning reconstruction frameworks further demonstrate that higher acceleration factors and near–real-time reconstruction are achievable without compromising diagnostic quality, directly addressing throughput limitations in clinical practice [53]. However, challenges related to robustness, vendor variability, and failure mode characterization remain important translational considerations.

#### 3.2.5. Clinical Translation, Validation and Limitations

Despite strong technical performance across segmentation, tissue characterization, and reconstruction, widespread clinical adoption of AI in CMR depends on rigorous external validation and integration into established workflows. Current evidence is largely derived from retrospective or single-center datasets, and prospective studies demonstrating incremental clinical benefit remain limited [45,54].

Key barriers include variability in acquisition protocols, dependence on large annotated datasets, and concerns regarding generalizability across institutions and scanner vendors. Furthermore, interpretability and clinician trust are essential for responsible deployment.

Overall, CMR represents one of the most advanced modalities for AI implementation in cardiovascular imaging. Automated cine-based segmentation appears technically mature, while tissue characterization and accelerated reconstruction continue to evolve. Sustained clinical impact will require multicenter validation, standardized evaluation frameworks, and outcome-driven studies to ensure safe and equitable integration into practice.

### 3.3. Cardiac Computed Tomography (CT)

#### 3.3.1. Automated Coronary Segmentation and Plaque Quantification

Artificial intelligence has substantially enhanced anatomical assessment in coronary computed tomography angiography (CCTA) by enabling automated coronary segmentation and quantitative plaque characterization. Contemporary consensus recommendations emphasize a paradigm shift from qualitative stenosis grading toward standardized, quantitative evaluation of total plaque burden and composition [55]. Automated measurement of total plaque volume, non-calcified and low-attenuation components, and plaque distribution improves reproducibility and reduces inter-observer variability compared with manual interpretation. Deep learning–based quantitative plaque analysis has been increasingly evaluated in recent systematic and methodological studies [56].

Rather than relying on fixed population cutoffs, AI-supported frameworks increasingly incorporate age- and sex-adjusted reference distributions derived from large multicenter cohorts, supporting individualized risk stratification and precision prevention strategies [55]. Emerging radiomics approaches further refine plaque phenotyping by extracting high-dimensional features associated with adverse cardiovascular events, although harmonization across scanners and software platforms remains a prerequisite for broad clinical deployment [57].

#### 3.3.2. Functional Assessment: CT-Derived FFR and Ischemia Prediction

Machine learning and computational approaches for CT-derived fractional flow reserve have enabled functional assessment directly from coronary CT angiography datasets. Early computational and machine learning frameworks demonstrated the feasibility of estimating FFR non-invasively from coronary CT images [58]. Subsequent clinical studies confirmed the diagnostic performance of CT-derived FFR compared with invasive measurements, supporting the evolution of CCTA from purely anatomical imaging toward combined anatomical–functional evaluation [59].

Beyond computational fluid dynamics–based FFR_CT_, deep learning approaches enable scalable ischemia prediction directly from anatomical datasets, reducing reliance on complex segmentation workflows [60,61]. Radiomics-based machine learning models further integrate plaque morphology and myocardial texture features to improve identification of functionally significant disease [57].

Collectively, these developments illustrate the evolution of CCTA from a purely anatomical modality toward integrated anatomical–functional characterization. However, variability in acquisition protocols, training datasets, and external validation strategies continues to limit universal implementation.

#### 3.3.3. Risk Stratification and Outcomes

AI-driven integration of clinical variables and detailed CCTA-derived imaging features has demonstrated incremental prognostic value beyond conventional risk scores. Large multicenter registry analyses show that machine learning models incorporating plaque burden, composition, and distribution outperform traditional risk stratification tools for long-term mortality prediction [62].

In parallel, fully automated deep learning–based coronary artery calcium (CAC) scoring algorithms achieve high agreement with expert interpretation while dramatically reducing processing time [63]. Multicenter external validation confirms robust performance for opportunistic CAC assessment from both gated and non-gated chest CT examinations, expanding scalable cardiovascular risk screening [64].

Together, these findings position AI-enhanced CT as a powerful platform for quantitative phenotyping and precision prognostication.

#### 3.3.4. AI in Aortic Root Assessment and Structural Heart Planning

Beyond coronary artery evaluation, artificial intelligence applications in cardiac CT increasingly extend to structural heart disease and transcatheter intervention planning. Accurate assessment of the aortic annulus, sinus of Valsalva dimensions, coronary ostial height, and ascending aorta geometry is critical for transcatheter aortic valve implantation (TAVI). Deep learning–based automated segmentation frameworks enable reproducible extraction of annular dimensions and root geometry directly from pre-procedural CT datasets, substantially reducing operator dependency and post-processing time.

Recent studies have demonstrated that fully automated CT analysis pipelines can achieve measurement accuracy comparable to expert manual assessment while providing standardized annulus sizing, implantation angle determination, and geometric modeling for procedural planning. For example, Arsalan et al. reported a deep learning–based algorithm for fully automated pre-procedural TAVI planning that enabled reliable extraction of aortic root parameters with high agreement to expert measurements, supporting workflow acceleration and reproducibility in structural heart interventions [65].

Beyond geometric assessment, emerging AI models are being developed to assist in prosthesis selection, predict paravalvular leak risk, and anticipate conduction disturbances after TAVI, illustrating a shift from pure anatomical automation toward decision-support systems. As global TAVI volumes continue to expand, AI-assisted structural CT analysis represents one of the most clinically translational domains within cardiovascular imaging.

While selected CT-based AI tools for coronary quantification and functional assessment have already achieved regulatory clearance, broader validation of automated structural planning frameworks across diverse valve platforms, scanner vendors, and anatomical phenotypes remains necessary to ensure consistent real-world performance [66,67].

#### 3.3.5. Limitations, Validation and Clinical Translation

Despite substantial technical progress, widespread clinical adoption of AI in cardiac CT requires rigorous external validation, harmonization across vendors, and demonstration of incremental clinical benefit. Variability in acquisition protocols, reconstruction algorithms, and population characteristics may affect generalizability across healthcare systems.

Recent perspectives emphasize that successful translation depends not only on algorithmic accuracy but also on workflow integration, interpretability, and standardized reporting frameworks [66,67]. While anatomical quantification and CAC automation appear relatively mature, decision-altering systems, particularly those integrating functional prediction and structural planning, require continued prospective validation.

### 3.4. Nuclear Cardiology (SPECT/PET)

Artificial intelligence (AI) is increasingly reshaping nuclear cardiology, particularly myocardial perfusion imaging (MPI) using single-photon emission computed tomography (SPECT) and positron emission tomography (PET). Contemporary reviews emphasize that AI applications in nuclear cardiology have evolved from early rule-based systems toward data-driven machine learning and deep learning frameworks capable of integrating imaging, clinical, and attenuation-correction information into unified diagnostic and prognostic models [68,69]. Rather than replacing expert interpretation, these systems function as quantitative augmentation tools that improve reproducibility, automate workflow-intensive tasks, and enhance risk stratification.

#### 3.4.1. Automated Diagnosis and Quantitative Perfusion Analysis

Deep learning approaches applied to SPECT MPI have demonstrated robust diagnostic performance for detecting obstructive coronary artery disease (CAD). In a comprehensive study, Hajianfar et al. developed an AI-powered framework using SPECT polar maps and hybrid supervision combining expert annotations and invasive coronary angiography data. The model achieved near–expert-level performance, with area under the ROC curve values approaching 0.89, while saliency mapping enhanced interpretability by highlighting clinically relevant perfusion defects [70].

These findings illustrate a broader trend described in recent reviews: AI improves consistency and reduces inter-observer variability by extracting complex spatial patterns beyond conventional perfusion defect metrics such as total perfusion deficit (TPD) or summed scores [68,69]. Importantly, gains in diagnostic performance tend to be incremental rather than disruptive, positioning AI as a decision-support enhancement rather than a replacement for established quantitative software.

In PET imaging, automated quantitative analysis has long been a strength of nuclear cardiology, and AI further refines this capability. Nakazato et al. demonstrated that automated three-dimensional Rb-82 PET/CT perfusion quantification achieved strong diagnostic accuracy against invasive coronary angiography (AUC ≈ 0.86), while generating reproducible perfusion and functional metrics without manual intervention [71]. Such fully automated pipelines illustrate how AI-enhanced PET can deliver scalable, standardized functional assessment across institutions.

#### 3.4.2. Prognostic Modeling and Risk Prediction

Beyond diagnostic classification, AI has shown particular promise in outcome prediction. Reviews of AI in nuclear cardiology highlight the ability of machine learning models to combine MPI-derived quantitative features with clinical variables and attenuation-correction CT data, improving prediction of major adverse cardiac events compared with perfusion metrics alone [68,69].

More broadly, systematic evaluations of machine learning in coronary artery disease demonstrate that integrative models incorporating imaging biomarkers consistently outperform traditional risk scores in outcome prediction, underscoring the conceptual framework within which nuclear AI operates [72]. While many of these studies are not modality-specific, they support the principle that combining quantitative imaging data with clinical variables enhances prognostic discrimination, a paradigm increasingly applied in SPECT and PET registries.

#### 3.4.3. Limitations and Clinical Translation

Despite encouraging developments, several limitations temper widespread adoption. Improvements in diagnostic metrics are often moderate, and performance varies across scanners, reconstruction algorithms, and patient populations. Reviews emphasize persistent challenges including limited availability of large, balanced training datasets, heterogeneity in reference standards, domain shift across institutions, and the need for explainable AI outputs to ensure clinician trust [68,69].

Importantly, most AI applications in nuclear cardiology have been validated retrospectively. Prospective studies demonstrating incremental clinical benefit over established quantitative nuclear software remain limited. Thus, while AI enhances automation, reproducibility, and risk stratification in SPECT and PET imaging, further multicenter validation and implementation-focused research are essential before routine clinical deployment.

### 3.5. Multimodality and Integrative Clinical Workflows

The integration of artificial intelligence (AI) across multimodal cardiovascular data represents a transition from modality-specific automation toward disease-centered decision-support systems. Contemporary cardiovascular care generates heterogeneous data streams including echocardiography, CT, CMR, nuclear imaging, electrocardiography, biomarkers, and electronic health records which AI can integrate into unified predictive frameworks. Such integrative models enable longitudinal disease modeling, improved risk stratification, and more comprehensive phenotyping than any single modality alone [73].

Multimodality imaging is particularly well suited for AI integration because each modality contributes complementary information: echocardiography provides dynamic functional assessment, CT detailed anatomical and coronary characterization, CMR tissue-level and volumetric quantification, and nuclear imaging perfusion and metabolic insights. AI-driven fusion of these structural, functional, and clinical features facilitates more precise disease characterization and supports personalized management strategies.

Cardiac amyloidosis illustrates the clinical potential of this approach. Machine learning models combining electrocardiographic patterns, echocardiographic strain metrics, CMR tissue characterization, and nuclear scintigraphy findings have demonstrated improved diagnostic discrimination compared with single-modality strategies [74,75,76]. In parallel, AI-based screening algorithms applied to ECGs or echocardiographic videos may enable earlier identification of high-risk patients and guide targeted downstream multimodality imaging [77].

Collectively, multimodal AI workflows represent an evolving frontier in cardiovascular imaging, shifting the paradigm from isolated quantitative tools toward integrated clinical decision-support ecosystems.

**Figure 3:**
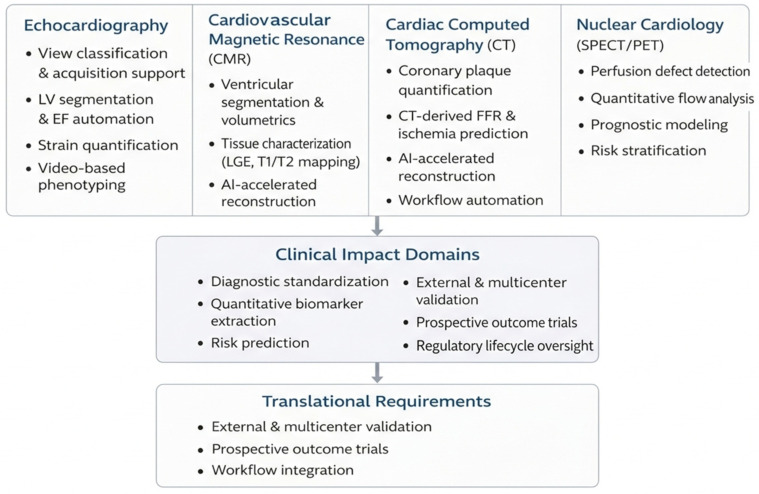
Artificial Intelligence Across Cardiovascular Imaging: Clinical Applications and Translational Framework.

The principal clinical applications of AI across major cardiovascular imaging modalities and their integration into multimodal workflows are summarized in Figure 3.

To provide a structured overview of clinically relevant AI use-cases across cardiovascular imaging modalities, Table 1 summarizes representative applications, typical model architectures, outputs, and their potential clinical value.

### 3.6. Bias, Fairness, and Generalizability in AI-Driven Cardiovascular Imaging

Despite strong technical performance across modalities, AI systems in cardiovascular imaging remain vulnerable to bias and limited generalizability. Cardiovascular imaging is characterized by heterogeneity in acquisition protocols, operator dependency (particularly in echocardiography), vendor-specific reconstruction algorithms, and variable disease prevalence, all of which increase the risk of domain shift when models trained in one environment are deployed in another [78].

Emerging evidence indicates that segmentation and quantification models may exhibit performance variability across demographic subgroups, including sex and racial categories, particularly when training datasets lack adequate representation [79]. Although most extensively studied in CMR, similar concerns apply across echocardiography, CT, and nuclear imaging.

Institutional variability further complicates implementation. Models trained on single-center datasets may experience performance degradation during external validation due to differences in hardware, acquisition protocols, and clinical practice patterns [80]. Multicenter training strategies and rigorous external validation are therefore essential before routine deployment.

Interpretability is equally critical for clinical adoption. While deep learning models may achieve high predictive accuracy, opaque decision pathways can limit clinician trust. Reporting frameworks such as CONSORT-AI and TRIPOD-AI provide guidance for transparent evaluation and validation of AI-driven clinical prediction models, supporting reproducibility and responsible implementation [81,82].

### 3.7. Comparative Maturity and Translational Readiness Across Modalities

Across cardiovascular imaging modalities, artificial intelligence has reached heterogeneous stages of technical maturity and clinical readiness. Echocardiography represents the most mature domain for workflow-level AI automation, particularly in view classification, ventricular segmentation, and ejection fraction estimation [19,42,44].

Cardiovascular magnetic resonance demonstrates strong technical performance in segmentation and reconstruction tasks, although large-scale prospective outcome validation remains limited [46,47,52]. In cardiac computed tomography, AI applications extend beyond anatomical quantification toward functional assessment and prognostic modeling, supporting increasingly integrated clinical decision-making [55,62].

In nuclear cardiology, AI has produced incremental improvements in diagnostic performance and risk stratification, primarily enhancing quantitative interpretation rather than fundamentally altering established workflows [68,69,70].

Multimodality AI frameworks represent an emerging frontier aimed at integrating structural, functional, and clinical data into unified disease-centered models. However, these approaches remain dependent on harmonized datasets, external validation, and effective workflow integration.

Overall, AI applications that automate established quantitative tasks (e.g., ventricular segmentation, ejection fraction estimation, coronary calcium scoring) demonstrate the highest level of maturity and external validation, whereas multimodal integration and advanced prognostic modeling remain dependent on prospective validation and clinical implementation strategies [73,74,75,76,77].

## 4. Limitations and Challenges in AI-Driven Cardiovascular Imaging

Despite rapid expansion of artificial intelligence in cardiovascular imaging, widespread clinical adoption remains limited by methodological and system-level barriers.

### 4.1. Data Bias and Limited Generalizability

Most AI models are trained on retrospective single-center datasets derived from relatively homogeneous populations and standardized acquisition protocols. These datasets often fail to capture real-world variability in age, sex, ethnicity, comorbidities, scanner vendors, and imaging parameters. Consequently, models demonstrating strong internal performance may degrade when applied to external populations due to dataset shift and demographic imbalance. Lack of robust multicenter and multi-vendor validation therefore remains a major barrier to clinical translation. Prospective pragmatic evaluations are also rare, and few AI tools have undergone outcome-driven trials reflecting routine clinical practice [37,39,46,54,79,83].

### 4.2. Interpretability and Medico-Legal Accountability

Although deep learning architectures achieve high diagnostic accuracy, their decision pathways often remain opaque. Saliency maps and attention mechanisms offer partial explanations but may not align with clinical reasoning. In cardiovascular imaging where results frequently guide high-risk interventions—limited interpretability raises concerns regarding error detection, accountability, and medico-legal responsibility. Transparent outputs and human-in-the-loop frameworks are therefore essential for safe implementation [18,38,39].

### 4.3. Workflow Integration and Infrastructure Constraints

Technical performance alone does not ensure clinical adoption. Many AI tools remain confined to research environments due to limited interoperability with PACS, RIS, EHR systems, and reporting platforms. Poor interface design may increase cognitive load rather than reduce it. Additionally, limited clinician training, insufficient IT infrastructure, and uncertain reimbursement pathways hinder implementation outside tertiary centers [37,67,84,85,86]. This contributes to an “elite-center phenomenon,” where AI systems are developed and validated in academic institutions but rarely deployed in community settings.

### 4.4. Regulatory and Lifecycle Challenges

Regulatory frameworks for adaptive AI systems remain evolving. Traditional static approval models may be insufficient for algorithms requiring continuous retraining and updating. Post-market surveillance, performance drift monitoring, and governance of adaptive learning systems are not yet standardized. Ethical issues related to data privacy, informed consent, and secondary data use further complicate large-scale deployment [37,38,39,66,87]. Without lifecycle-based oversight, AI systems risk becoming static products rather than continuously improving clinical tools.

### 4.5. Limited Evidence of Patient-Centered Benefit

Improvements in technical metrics such as AUC or Dice coefficient do not necessarily translate into better clinical outcomes. Few studies have demonstrated reductions in adverse events, improved therapeutic decisions, decreased downstream testing, or survival benefit attributable to AI-assisted imaging. Large-scale randomized and pragmatic implementation trials are therefore required to establish cost-effectiveness and real-world clinical impact [12,17,37,38,39].

### 4.6. From Prototypes to Sustainable Ecosystems

Despite thousands of publications, relatively few AI algorithms have achieved durable large-scale clinical deployment. Many remain proof-of-concept tools developed on curated datasets without continuous retraining strategies, multicenter governance, or federated learning infrastructures. Future progress will depend on transitioning from isolated academic prototypes toward collaborative, continuously monitored ecosystems that include tertiary and community centers. Transparent validation, standardized update pipelines, lifecycle regulatory oversight, and alignment with reimbursement models will determine whether AI becomes a routine clinical partner or remains confined to specialized environments [37,67].

**Figure 4:**
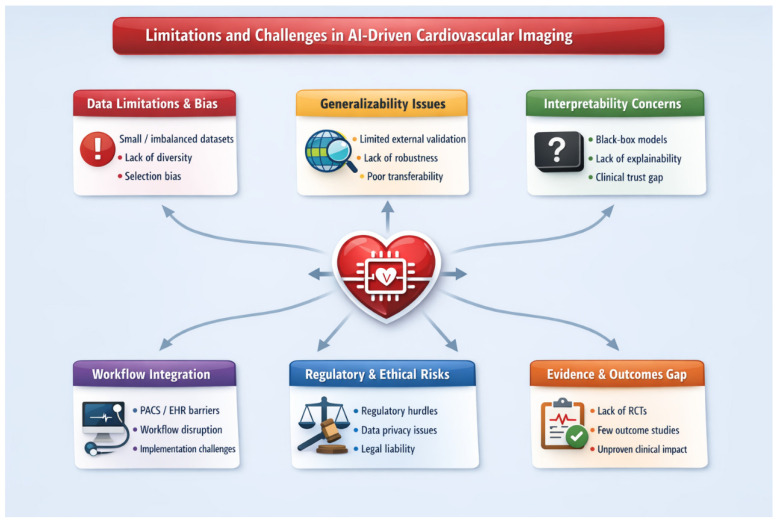
Key limitations and challenges in the clinical translation of artificial intelligence in cardiovascular imaging.

The major limitations and challenges affecting the clinical translation of artificial intelligence in cardiovascular imaging are summarized in Figure 4.

## 5. Future Directions

Future progress in artificial intelligence for cardiovascular imaging will depend on demonstrating meaningful patient-centered clinical impact. High algorithmic performance does not inherently translate into improved diagnosis or outcomes. The next phase of development therefore requires pragmatic clinical trials, workflow-integrated randomized studies, and health-economic analyses evaluating endpoints such as reduction of diagnostic error, optimization of therapeutic decision-making, avoidance of unnecessary invasive procedures, and ultimately improvement in morbidity and mortality [88,89].

AI in cardiology is increasingly evolving from isolated imaging algorithms toward integrated decision-support systems embedded within routine clinical practice. Beyond image analysis, generative AI tools such as large language models may support clinical documentation, literature synthesis, and guideline navigation, potentially improving workflow efficiency and clinician–AI interaction. However, responsible integration requires robust validation, governance frameworks, and clear clinical accountability [90].

Another critical direction involves moving from isolated image-based models to integrated, longitudinal disease modeling. Cardiovascular pathology reflects complex interactions between imaging findings, clinical variables, electrocardiographic signals, biomarkers, genetics, and environmental factors. Multimodal deep learning architectures capable of integrating heterogeneous data streams may enable earlier detection of disease progression, improved risk stratification, and individualized therapeutic monitoring. Achieving this vision will require interoperable data infrastructures, standardized data representations, and robust multicenter validation [91,92].

Human–AI collaboration will remain central to clinical deployment. Human-in-the-loop frameworks incorporating explainability tools, uncertainty quantification, and confidence-aware outputs can preserve clinician oversight while improving trust and interpretability. Continuous clinician feedback and adaptive learning strategies may further enhance robustness in real-world clinical environments [93,94].

Standardized evaluation and reporting frameworks are essential to ensure reproducibility and comparability across studies. Guidelines such as TRIPOD-AI, CONSORT-AI, and SPIRIT-AI represent important steps toward improving methodological rigor and transparency, while multicenter validation across vendors and populations will be necessary to mitigate bias and ensure generalizability [81,95].

Collaborative data infrastructures will also play a key role in future development. Privacy-preserving approaches including federated learning and swarm learning enable multicenter model training without centralized data sharing, although challenges related to data heterogeneity, governance, and model convergence remain [96,97].

As AI systems become more adaptive, regulatory frameworks must evolve toward lifecycle-based oversight and real-world performance monitoring. Professional societies will be instrumental in defining validation standards, certification requirements, and guidance for clinical implementation [98,99].

Finally, sustainable integration of AI will depend on workforce readiness. Imaging specialists must develop foundational AI literacy to critically interpret algorithmic outputs and participate in model governance. Rather than replacing clinicians, AI is likely to redefine the cardiovascular imager’s role toward integrative interpretation and oversight of automated systems [100,101].

Overall, the future of cardiovascular imaging AI will depend less on incremental algorithmic improvements and more on scalable validation, inclusive multicenter collaboration, and clinically embedded deployment strategies. Establishing global collaborative networks that include peripheral and community centers may promote equitable access to AI-enhanced imaging and prevent concentration of innovation within specialized institutions [102].

**Figure 5:**
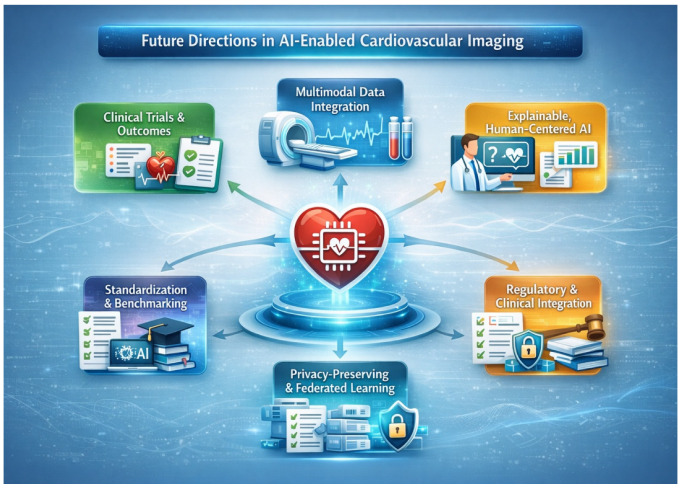
Future directions of artificial intelligence in cardiovascular imaging.

Figure 5 summarizes the key future directions for artificial intelligence in cardiovascular imaging, highlighting the transition toward patient-centered outcomes, multimodal data integration, human–AI collaboration, standardized validation, and regulatory and educational frameworks required for clinical translation.

## 6. Conclusions

Artificial intelligence is reshaping cardiovascular imaging, although translational maturity varies across modalities.

In echocardiography, AI-based view classification, automated ventricular segmentation, and ejection fraction estimation are already integrated into commercial platforms and improve reproducibility in routine practice.

In cardiac CT, automated coronary calcium scoring and quantitative plaque assessment are clinically implemented in several centers, while CT-derived FFR and structural heart planning tools continue to expand with ongoing validation.

In cardiovascular magnetic resonance, automated cine segmentation and AI-driven reconstruction demonstrate high technical maturity and support workflow acceleration, although prospective outcome-based validation remains limited.

In nuclear cardiology, AI primarily enhances quantitative perfusion analysis and risk stratification, functioning as decision-support augmentation rather than replacing expert interpretation.

Overall, cardiovascular imaging is transitioning from modality-specific automation toward integrated clinical decision-support systems. Sustainable implementation will depend on multicenter validation, regulatory oversight, bias mitigation, clinician engagement, and demonstration of patient-centered benefit.

When embedded within transparent and continuously monitored clinical workflows, AI has the potential to improve efficiency, reproducibility, and precision in cardiovascular imaging.

## Figures and Tables

**Table 1:** Representative clinical AI applications across cardiovascular imaging modalities.

Modality	AI Application	Clinical Target	Evidence Level	External Validation	Main Failure Modes	Implementation Stage
Echo- cardiography	View classification	Automated acquisition support	Multicenter retrospective	Yes (limited vendor diversity)	Atypical anatomy, poor acoustic window	Research/Early clinical
	LV segmentation & EF estimation	Quantitative LV function	Prospective randomized + multicenter	Yes	Arrhythmia, image noise, LV aneurysm	CE/FDA-cleared tools
	Video-based prognostic modeling	HF, AF, mortality prediction	Retrospective multicenter	Limited	Dataset shift, demographic bias	Research
CMR	Cine segmentation (LV/RV volumes)	Automated volumetrics	Multicenter benchmarking	Yes (multi-vendor variable)	Basal/apical slice mislabeling, RV contour errors	Early clinical integration
	LGE/T1–T2 mapping segmentation	Tissue characterization	Retrospective multicenter	Limited prospec-tive	Scar border misclassifi-cation, artifacts	Research/Emerging
	DL reconstruction	Acceleration & artifact reduction	Technical + feasibility studies	Limited	Instability at high acceleration	Vendor-integrated
Cardiac CT	Coronary segmentation & plaque quantification	Atherosclerosis phenotyping	Multicenter registries	Yes	Heavy calcification, motion artifacts	CE/FDA-cleared
	CT-derived FFR	Functional stenosis assessment	Multicenter prospective trials	Yes	Severe calcification, segmentation errors	Commercial platforms
	Automated CAC scoring	Risk screening	Multicenter external	Yes	Non-gated CT variability	CE/FDA-cleared
	Structural CT (TAVI planning)	Annulus sizing & geometry	Retrospective + early prospective	Limited	Severe calcification, bicuspid anatomy	Early adoption
Nuclear (SPECT/PET)	Automated perfusion analysis	CAD detection	Retrospective multicenter	Limited	Attenuation artifacts	Vendor-assisted
	ML-based prognostic models	MACE prediction	Registry-based retrospective	Limited	Population shift	Research

## Data Availability

No new data were created or analyzed in this study.

## Abbreviations

The following abbreviations are used in this manuscript:

AI	Artificial Intelligence
CMR	Cardiovascular Magnetic Resonance
CT	Computed Tomography
MRI	Magnetic Resonance Imaging
PET	Positron Emission Tomography
LVEF	Left Ventricular Ejection Fraction
EF	Ejection Fraction
Auto-EF	Automated Ejection Fraction
M-mode	Motion mode
ICC	Intraclass Correlation Coefficient
ML	Machine Learning
DL	Deep Learning
CNNs/CNN	Convolutional Neural Networks
RNN	Recurrent Neural Network
LSTM	Long Short-Term Memory
CCTA	Coronary Computed Tomography Angiography
FFR-CT/CT-FFR	Fractional Flow Reserve derived from CT
FFR	Fractional Flow Reserve
ROC	Receiver Operating Characteristic
AUC	Area Under the Curve
EDV	End-Diastolic Volume
ESV	End-Systolic Volume
GLS	Global Longitudinal Strain
LV	Left Ventricle
RV	Right Ventricle
LGE	Late Gadolinium Enhancement
ECV	Extracellular Volume
SENSE	Sensitivity Encoding
GRAPPA	Generalized Autocalibrating Partial Parallel Acquisition
MoDL	Model-based Deep Learning
RAKI	Robust Artificial-neural-networks for K-space Interpolation
DeepSPIRiT	Deep learning variant of SPIRiT reconstruction
NHS	National Health Service
ACDC	Automatic Cardiac Diagnosis Challenge
STEMI	ST-Elevation Myocardial Infarction
PACS	Picture Archiving and Communication System
RIS	Radiology Information System
EHR/EHRs	Electronic Health Record(s)
MPI	Myocardial Perfusion Imaging
SPECT	Single-Photon Emission Computed Tomography
CAD	Coronary Artery Disease
MACE	Major Adverse Cardiac Events
ECG	Electrocardiogram
ATTR-CM	Transthyretin Amyloid Cardiomyopathy
ATTR	Transthyretin
AL	Amyloid Light-chain
QCI	Quantitative Cardiovascular Imaging
